# Myths and misconceptions about tuberculosis transmission in Ghana

**DOI:** 10.1186/1472-698X-13-38

**Published:** 2013-09-12

**Authors:** Joshua Amo-Adjei, Akwasi Kumi-Kyereme

**Affiliations:** 1 Department of Population and Health, University of Cape Coast, Cape Coast, Ghana

**Keywords:** Tuberculosis, Myths, Misconceptions transmission, Spatial, Ghana

## Abstract

**Background:**

Myths and misconceptions about TB can serve as a barrier to efforts at reducing stigmatisation of people infected and affected by the disease. Understanding such drivers of myths and misconceptions is important for improving information, education and communication (IEC) efforts of national control and preventive interventions. This study therefore assesses the influence of interaction of spatial, socioeconomic and demographic characteristics on myths and misconceptions.

**Methods:**

Data was drawn from male (N = 4,546) and female (N = 4,916) files of the 2008 Ghana Demographic and Health Survey. A myth and misconception variable was created from five-related constructs with internal consistency score of r = 0. 8802 for males (inter-item correlation: 0.5951) and for females, r = 0. 0.9312 (inter-item correlation: 0.7303). The Pearson Chi-square was used to test the bivariate relationship between the independent variables and the dependent variable. Logistic regression was subsequently used to explore the factors determining myths and misconceptions of TB transmission.

**Results:**

Majority of Ghanaians (males: 66.75%; females: 66.13%) did not hold myths and misconceptions about TB transmission. Females resident in the Upper East (aOR = 0.31, CI = 0.17-0.55) and Upper West (aOR = 0.41, CI = 0.24-0.69) and males resident in the Northern (aOR = 0.23, CI = 0.13-0.39) and the Greater Accra (aOR = 0.25, CI = 0.16-0.39) regions were independently associated with no misconceptions about TB transmission. Significant differences were also found in education, ethnicity and age.

**Conclusion:**

That spatial and other socioeconomic difference exists in myths and misconceptions suggest the need for spatial, socioeconomic and demographic segmentations in IEC on TB. This holds potentials for reaching out to those who are in critical need of information and education on the transmission processes of TB.

## Background

In 1993, tuberculosis (TB) was declared a global emergency and the estimated number of infections was about 70 million with 1.6 million deaths. Between 2007 and 2009, there was approximately one per cent increase in global TB incident cases: from 9.3 million incident cases to 9.4 million (139/100,000). Of the estimates in 2009, 55 per cent was reported in Asia, 30 per cent in Africa. In country specific terms, India (1.6–2.4 million), China (1.0–1.6 million), South Africa (0.38–0.57 million), Nigeria (0.37–0.55 million) and Indonesia (0.34–0.52 million) were the countries with the highest burden of TB around the world in 2008 [1-3].

In the last decade, efforts to reduce the lethal effects of TB have been intensified through concerted global actions (e.g. the Millennium Development Goals, goal 6). However, the existing control situation in Ghana and other developing countries portend threat to achieving TB-related MDGs. This is notwithstanding the positive signal that ‘halting and reversing the spread of TB’ has been achieved at the global level [4].

In Africa, incidence is still rising, apparently, as a result of human immune virus and acquired immune deficiency syndrome (HIV/AIDS). Out of the present twenty-two high burden countries, eight are in Africa (Kenya, South Africa, Zimbabwe, Ethiopia, Nigeria, Democratic Republic of Congo, Uganda and Mozambique). In these countries where TB is quite prevalent, the rates of infection are generally parallel with HIV/AIDS rates [5].

Whereas Ghana is not one of the high burden countries, historical evidence on TB suggests that there is the need for continuous surveillance of the disease to avoid sudden epidemics. Present estimates of TB case detection levels in Ghana suggest a decline from 40 per cent to 36 per cent between 2003 and 2007, while treatment success ranged from around 60 per cent in 2002 to 76 per cent in 2006. Of the estimated 47,632 TB cases in 2010 in the country, only 15,145 were detected (of both relapsed and new cases). The proportion of deaths among the total cases notified was about 8.7 per 100,000 people. The situation becomes troubling when gauged against the fatal effects of co-morbidity of HIV and TB. Put together, HIV/AIDS and TB accounted for 7% of deaths in all age groups, the second highest to malaria (13%) in 2009 [6].

One prime challenge to TB control activities is myths and misconceptions about the disease [7]. These negative dispositions often result in delayed health seeking as well as stigmatisation of people infected. For instance, Peltzer, Mngqundaniso and Petros [7] found among a South African sample that smoking, mosquito bites etc. were considered to be causes of TB and these hindered seeking early treatment. In rural Ethiopia, exposure to cold air was reported to be a cause of TB infection. The impacts of such misconceptions and myths, according to past studies [8-16] usually result in concealment of status, fear, and shame, which do not promote treatment behaviours.

Presently, the worldwide strategy, Stop TB, is the general framework within which TB is being fought in Ghana [17]. The strategy recognises that individuals and communities infected and affected by TB have to be empowered with critical mass of information on TB transmission, availability of cure, status disclosure, community participation in TB control etc. It is believed that the availability and utilisation of such information will have positive influence on stigmatisation, early treatment seeking behaviour and other important inputs into TB control [17]. Khandokor et al. (2011) found spatial differences in myths and misconceptions about TB and further contended that these differences could be accounted for by socioeconomic disparities. Thus, individuals in certain locations may not be served with the adequate information about specific diseases such as TB. The spatial differences could however, be moderated by factors such as age, education, ethnicity, marital status, gender, exposure to media (print, radio, television, etc.) religion amongst others. Our aim here is therefore to assess the spatial, socioeconomic and demographic drivers of myths and misconceptions of TB transmission in Ghana. Findings of the study are expected to make useful contributions to information, education and communication strategies for dispelling myths and misconceptions about TB in Ghana.

The focus on myths and misconceptions is borne out of the fact that they are potential threat to preventive efforts [18-20]. Although the various studies by Dodor et al. add to our understanding of certain aspects of myths and misconceptions, the contribution of this study is that it uses a nationally representative data to examine the phenomenon, thereby providing clues about characteristics of people who are likely to hold misconceptions. Another important contribution of this paper is bridging the literature gap between the biomedical base and the social science discourses on TB in Ghana. Amo-Adjei [21] shows that more than 70% of studies on TB between 1947 and 2010 in Ghana focused on the biomedical aspects of the disease. Gandy and Zumla [22] have criticized such approach to TB research where disproportionate emphasis is placed on biomedical paradigm. Recently, Lonnroth et al. [23] have argued for an approach to TB research that combines biomedical and sociocultural paradigms in order to achieve greater impacts on control measures.

## Methods

### Data

We draw on the 2008 Ghana Demographic and Health Survey, a household survey based on probability sample of 11,778 households across the country for this study. Eligible females and males in half of the selected households were interviewed. Among females, those eligible were between 15–49 years and among males, the range was 15–59 years. MEASURE DHS administered the survey in collaboration with Ghana Statistical Service (GSS) and Ghana Health Service (GHS). The survey was conducted between September and November 2008. A multi-staged sampling was used for the survey. The first stage of the sampling involved a selection of 412 clusters based on updated enumeration areas of the 2000 Population and Housing Census. In the second stage of sampling, households were randomly selected from clusters selected at the first level. The survey in 2008 was the first time information on TB was collected. This was intended as one of the preparatory works towards a national TB prevalence survey to be conducted by the National TB Control Programme of Ghana. The total female respondents were 4,916 and 4,546 male respondents and the corresponding response rates for females and males were 97% and 96% respectively. MEASURE DHS gave permission for the use of the data (http://www.measuredhs.com).

### Dependent variable

With respect to the main dependent variable, five related questions on TB transmission were collapsed into a single binary outcome variable. The questions were TB spreads by: (1) sharing utensils [no = 0; yes = 1], (2) touching [no = 0; yes = 1], (3) sexual contact [no = 0; yes = 1], (4) mosquito bite [no = 0; yes = 1] and (5) through food [no = 0; yes = 1]. Responses to these five items were coded (“Yes”, “No”) and used to construct an index to tap into respondents’ myths and misconceptions about TB transmission. A respondent was considered to hold myths and misconceptions if he/she answered yes to at least one of the questions while those who answered ‘no’ to all the five items were considered not hold myths and misconceptions. The resulting variable was coded “no myths and misconception” = 0 and “myths and misconception” = 1. In the context of this study, myths and misconceptions are defined as beliefs or opinions about a particular phenomenon that are commonly held to be true but which are, indeed, erroneous [24]. Standardised Cronbach alpha reliability test on the dependent variable returned the following results for males (r = 0. 8802; inter-item correlation: 0.5951) and for females (r = 0. 0.9312; average inter-item correlation: 0.7303) respectively.

### Independent variables

The selection of the following independent variables are in consonance with past studies [25,26] and these are region of residence (Western = 1; Central = 2; Greater Accra = 3; Volta = 4; Eastern = 5; Ashanti = 6; Brong-Ahafo = 7; Northern = 8; Upper East = 9; Upper West = 10), education (no education = 0; primary education = 1; secondary education = 2; higher education = 3), wealth status (poorest/poorer = 1; middle = 2; richer/richest = 3), age of respondents (15-19 = 1; 20-24 = 2; 25-29 = 3; 30-34 = 4; 35-39 = 5; 40-44 = 6; 45-49 = 7; 50-54 = 8; 55-59 = 9), marital status (never married = 0; currently married = 1; formerly married = 2), rural/urban residence (urban = 0; rural = 1), ethnicity (Akan = 1; Ga/Adangbe = 2; Ewe = 3; Mole/Dagbani = 4; Others groups = 5), religious denomination of respondents (Catholic = 1; Protestant = 2; Protestant/Charismatic = 3; Moslem = 4; Traditional/Others = 5), exposure to radio (no exposure = 0; moderate = 1; high exposure = 2), exposure to print (no exposure = 0; moderate = 1; high exposure = 2) and exposure to television (no exposure = 0; moderate = 1; high exposure = 2). The household wealth index is originally a five-point outcome (poorest, poorer, middle, richer and richest) of aggregate wealth of a household derived from possession of cars, agricultural land, television, bicycle, motorcycle, and a host of other consumer durable goods. We collapsed the five into three because initial test run of the data did not show any significant differences between the lower and upper two categories.

### Statistical analysis

First, the Pearson Chi-square test was used to compute the bivariate association between each of the independent variables and the dependent variables with associations being at significant 95%. Second, bivariate logistic regression analysis of region of residence with the misconception variable was conducted. Following this, we adjusted for the effects of the other independent variables described in the preceding section. Separate analyses were conducted for males and females. This was due to the absence of unique identifiers that allow merging of female and male files of DHS. The logistic regression results are reported in odds ratio (OR) and significant at 95% CI. No evidence of multicollinearity among the independent variables was observed in the initial diagnostic tests. All the analyses were weighted and this was done to control for the effect of over and under sampling which are associated with national surveys. All statistical analyses were conducted with STATA 12.0 (College Station, Texas 77845 USA).

**Table 1:** Myths and misconceptions about TB transmission by background characteristics

**Males**			**Females**	
**Factors**	**N**	**%**	**N**	**%**
Region	227.64(0.000)^*^		374(0.000)^*^	
Western	478	63.98	447	48.07
Central	376	45.94	423	65.62
Greater Accra	734	75.96	825	64.79
Volta	418	57.57	430	61.39
Eastern	470	60.96	483	73.83
Ashanti	857	67.07	1,010	64.36
Brong-Ahafo	384	75.01	425	74.46
Northern	477	82.89	466	85.14
Upper East	249	67.66	254	90.26
Upper West	120	46.70	122	89.12
Residence	0.1742(0.676)		30.39(0.000)^*^	
Urban	2,021	42.58	2,383	64.56
Rural	2,208	56.92	2,535	67.60
Religion	19.91(0.000)^*^		87.85(0.000)^*^	
Christian	1,419	63.16	1,439	69.03
Pentecostal	1,884	66.41	2,371	64.50
Moslem	757	72.01	738	76.66
Traditional	506	70.25	367	76.24
Ethnicity	26.92(0.000)^*^		257.55(0.000)^*^	
Akan	2,162	64.63	2,493	62.24
Ga/Dangme	298	74.64	343	68.19
Ewe	672	61.92	633	64.04
Mole-Dagbani	759	70.83	794	82.97
Others	674	70.31	651	79.50
Education	28.20(0.000)^*^		75.52(0.000)^*^	
No Schooling	639	74.43	1,041	76.56
Primary	660	69.74	987	69.05
Secondary+	3,255	64.57	2,882	65.50
Wealth index	6.53(0.038)^*^		100.38(0.000)^*^	
Poor/poorest	1,624	68.54	1,683	75.66
Middle	783	63.42	979	65.20
Rich/Richest	1,260	66.62	2,253	64.65
Marital status	59.16(0.000)^*^		61.16(0.000)^*^	
Never	1,942	73.36	1,593	75.61
Married	2,404	62.24	2,876	66.74
Formerly	221	57.77	446	54.77
Age cohort	105.93(0.000)^*^		135.87(0.000)^*^	
15–19	910	76.91	1,024	79.40
20–24	704	72.26	877	76.51
25–29	623	69.55	832	67.05
30–34	532	66.83	644	65.54
35–39	528	62.60	638	61.47
40–44	393	56.13	470	57.55
45–49	363	59.26	428	56.08
50–54	297	53.10	—	—
55–59	212	58.53	—	—
Print media	12.47(0.002)^*^		25.29(0.000)^*^	
No exposure	2,737	68.62	3,750	69.39
Moderate	1,404	63.49	1,009	68.39
High	416	64.95	147	51.44
Radio	27.68(0.000)^*^		99.03(0.000)^*^	
No exposure	259	81.60	746	79.58
Moderate	971	67.49	1,611	70.23
High	3,332	65.35	2,554	64.50
Television	1.82(0.402)^*^		40.71(0.000)^*^	
No exposure	1,162	66.94	1,798	71.93
Moderate	1,620	67.13	1,441	67.68
High	1,779	66.71	1,670	65.68

^*^Chi-square significant at 5%. P-values indicated in brackets.

## Results

Overall, 66.75% of males and 66.13% of females did not hold misconceptions about TB transmission. In Table 1, a bivariate analysis of the relationships between the dependent and independent variables are presented for both females and males. The results (Table 1) showed that region of residence, educational level, religious affiliation, ethnicity, household wealth status, exposure to print, radio and television, marital status and age cohort were all associated with myths and misconceptions about TB transmission. Respondents from the Northern Region were less likely to hold myths and misconceptions about TB transmission compared to those from the other regions (Table 1). Although urban–rural residence was found to be associated with myths and misconceptions of TB transmission among women, no such associations were found from the males’ data.

**Table 2:** Multivariable logistic regression results on myths and misconceptions about TB transmission in Ghana

	**Males**				**Females**			
	**Model 1**	**95% CI**	**Model 2**	**95% CI**	**Model 3**	**95% CI**	**Model 4**	**95% CI**
	**Odds ratio**		**Adjusted odds ratio**		**Odds ratio**		**Adjusted odds ratio**	
Region^a^								
Western	0.483^***^	[0.357,0.653]	0.448^***^	[0.291,0.689]	1.796^**^	[1.222,2.639]	1.775^**^	[1.205,2.616]
Greater Accra	0.290^***^	[0.215,0.389]	0.257^***^	[0.166,0.397]	1.078	[0.718,1.617]	1.038	[0.651,1.656]
Volta	0.639^**^	[0.472,0.865]	0.613^*^	[0.398,0.945]	1.244	[0.816,1.897]	1.475	[0.890,2.444]
Eastern	0.508^***^	[0.376,0.685]	0.498^**^	[0.319,0.776]	0.723	[0.487,1.072]	0.672	[0.437,1.034]
Ashanti	0.447^***^	[0.338,0.592]	0.436^**^	[0.261,0.730]	1.109	[0.744,1.652]	1.090	[0.719,1.653]
Brong-Ahafo	0.282^***^	[0.202,0.393]	0.289^***^	[0.190,0.438]	0.685	[0.438,1.071]	0.753	[0.485,1.168]
Northern	0.202^***^	[0.147,0.278]	0.232^***^	[0.138,0.390]	0.319^***^	[0.200,0.509]	0.562^*^	[0.340,0.928]
Upper East	0.364^***^	[0.263,0.503]	0.414^**^	[0.239,0.717]	0.172^***^	[0.105,0.283]	0.308^***^	[0.171,0.556]
Upper West	0.973	[0.723,1.310]	1.152	[0.672,1.976]	0.238^***^	[0.151,0.374]	0.408^***^	[0.240,0.692]
Residence^b^								
Rural			0.983	[0.756,1.280]			1.122	[0.859,1.466]
Religion^c^								
Orthodox			1.222	[0.962,1.551]			1.032	[0.764,1.394]
Pentecostal/Charismatic			1.193	[0.938,1.516]			1.200	[0.895,1.610]
Moslem			0.997	[0.719,1.383]			1.314	[0.931,1.856]
Ethnicity^d^								
Akan			0.764^*^	[0.586,0.997]			1.537^**^	[1.143,2.066]
Ga-Dangme			0.544^**^	[0.365,0.812]			1.538^*^	[1.031,2.294]
Ewe			0.840	[0.615,1.145]			1.299	[0.877,1.926]
Mole-Dagbani			0.925	[0.672,1.272]			1.056	[0.764,1.459]
Education^e^								
Primary			1.406^**^	[1.106,1.788]			1.211	[0.972,1.509]
Secondary & higher			1.678^***^	[1.340,2.102]			1.390^**^	[1.102,1.754]
Wealth index^f^								
Middle			1.046	[0.813,1.344]			1.211	[0.957,1.533]
Richer/richest			1.024	[0.772,1.358]			1.128	[0.857,1.485]
Marital status^g^								
Married			0.994	[0.789,1.252]			0.959	[0.769,1.197]
Never married			1.098	[0.765,1.576]			1.177	[0.872,1.589]
Age cohort^h^								
20-24			1.310^*^	[1.042,1.648]			1.153	[0.904,1.470]
25-29			1.648^***^	[1.275,2.129]			1.807^***^	[1.363,2.396]
30-34			1.873^***^	[1.374,2.553]			2.234^***^	[1.652,3.022]
35-39			2.430^***^	[1.780,3.318]			2.518^***^	[1.888,3.359]
40-44			2.981^***^	[2.164,4.107]			2.894^***^	[2.078,4.030]
45-49			2.729^***^	[1.950,3.819]			2.950^***^	[2.087,4.171]
50-54			3.172^***^	[2.212,4.548]			–	–
55-59			2.410^***^	[1.600,3.629]			–	–
Print media^i^								
Moderate			1.145	[0.960,1.365]			1.004	[0.830,1.215]
High exposure			1.194	[0.905,1.575]			1.842^**^	[1.202,2.821]
Radio^j^								
Moderate			1.640^**^	[1.169,2.302]			1.556^***^	[1.217,1.988]
High exposure			1.477^*^	[1.041,2.094]			1.713^***^	[1.341,2.187]
Television^k^								
Moderate			0.917	[0.735,1.145]			1.090	[0.906,1.311]
High			1.001	[0.785,1.276]			1.052	[0.854,1.296]
_Cons	1.182	[0.937,1.490]	0.301^***^	[0.161,0.562]	0.583^***^	[0.428,0.794]	0.0808^***^	[0.0439,0.149]
Log lik.	-2838.3		-2724.9		-2854.6		-2724.9	
Chi-squared	228.8		306.9		207.8		436.8	
N	4568		4537		4916		4895	

^*^p?<?0.05, ^**^p?<?0.01, ^***^p?<?0.001.

*Base categories: Region (Central)*^*a*^*; Type of place of residence (Urban)*^*b*^*; Religion (Traditional/None)*^*c*^*; Ethnicity (Others)*^*d*^*; Education (No formal)*^*e*^*; Wealth (Poor/Poorest)*^*f*^*; Age (15–19)*^*g*^*; Marital status (Never married)*^*h*^*; Print media (No exposure)*^*i*^*; Radio (No exposure)*^*j*^*; Television (No exposure)*^*k*^.

The inferential statistics results shown in Table 2 indicate significant differences among the regions. Models 1–2 are bivariate and multivariate models for males while Models 3–4 (Table 2) are for females. Using Central Region as the reference category for both sexes, male respondents from the Eastern, Brong-Ahafo Northern, Upper East and Upper West Regions are significantly less likely to hold myths and misconceptions about TB transmission. We find females from the Western (significant at *p* < 0.01), Greater Accra, and Volta to be more likely to hold myths and misconceptions about TB transmission.

In Models 2 (males) and 4 (females) [Table 2], where the respective models are adjusted with socioeconomic and demographic factors, we find no substantial changes in the odds ratios. The magnitude of changes in the strength of effect is noticeable in both males and females (Table 2 Model 4). Particularly, for females in the Northern, Upper East and Upper West regions, the likelihood of rejecting myths and misconceptions increases slightly but still lower than the likelihood in the reference region (Central) as well as other regions (Western, Greater Accra, Volta and Ashanti). However, among males from the Western, Greater Accra, Volta, Eastern, Brong-Ahafo and Ashanti regions, the probability of holding myths and misconceptions about TB declines.

In terms of other socioeconomic variables and their relationship with myths and misconceptions, education provides very surprising results. We find significantly direct relationship between education and misconceptions about TB transmission – higher education corresponds to more chances of myths and misconceptions contrary to our expectations. However, in the DHS survey, a positive effect of education was observed on knowledge (whether one could transmit TB through coughing/sneezing or not) of TB transmission. To be sure that the effect of education on myths and misconceptions was not arising from the aggregation of the five constructs, each of them was regressed on education (data not shown). The results observed on the index of myths and misconceptions were confirmed by the analysis of each of the variables. Household wealth index also showed direct positive but insignificant relationship with misconception. Significant direct positive differences are found in misconceptions and age. That is, with increasing age, the propensity of entertaining misconceptions about TB rises. High exposure to almost all sources media included in this study showed positive, and sometimes, significant relationship with myths and misconceptions. The higher one is educated, the more likely the person is exposed to media channels. Given the positive relationship between education and myths and misconceptions on the one hand and the findings about education and exposure to radio, print and television on the other hand, there seem to be a confirmation of a kind.

## Discussion

Our interest in this paper was to examine spatial and other socioeconomic and demographic characteristics that drive myths and misconceptions about TB in Ghana. The study ties in with current efforts of the NTP-Ghana to increase awareness of the different facets of the disease. The variables used to construct myths and misconceptions also have important implications for stigma reduction efforts. Thus, when people are aware that TB cannot be transmitted through sharing utensils, food, sexual contact, physical touch etc. they are likely to be receptive to people infected with, and affected by TB. We find significant spatial, educational, age and ethnic differences in myths and misconceptions about TB.

We find significant spatial differences in myths and misconceptions about how TB is transmitted in Ghana. Khandoker et al. [25] argued that conceptions about TB could vary across spatial boarders, usually accounted for by differences in socioeconomic development. Among males, the differences are much pronounced than females. Controlling for other socioeconomic variables, females resident in the three northern regions (Northern, Uppers East and West) were found to have less likelihood of expressing myths and misconceptions about TB transmission. Assuming that myths and misconceptions were driven by differences in socioeconomic development as Khandoker et al. [25] posit, we had expected that residents in the three northern regions would be more inclined to express myths and misconceptions. However, what we find is contrary to Khandokor at al’s. It is difficult at this stage to show the specific factors that may be accounting for this pattern. Perhaps, a comparative qualitative study would throw more light on the contextual issues behind the numbers.

Contrary to expectation and evidence from other studies [26], higher education, particularly beyond secondary education did not show any significant association with low myths and misconceptions about TB transmission. This finding is a departure from many existing studies, particularly, in the light of allocative efficiency hypothesis [27]. Allocative efficiency hypothesis is based on assumption that higher education improves people’s views about certain health issues [27]. The emerging literature on this hitherto, generic assumption seems to challenge such generalisations. For example, Altindag et al. [28] reported that college or higher education did not necessarily improve views on issues that are of importance to public health. As Grossman [27] argued, there can always be a “third variable” such as the home environment, which can confound educational influence. In the light of the forgoing, there is a need for caution regarding the conventional one-size-fits-all assumption about education and beliefs about health matters.

Age was found to have significant negative influence on conceptions about TB. That is, the older cohorts were inclined to believe that TB could be transmitted through touching, sexual contacts, sharing utensils, mosquito bites and sharing food. The cohort effect may be attributed to generational differences in how myths and misconceptions about TB have evolved in the country. For instance, males between 55–59 years were born between 1949–53 while females between 45–49 years were born between 1959–63. The respective cohorts became adolescents in the 1960s and 70s when myths and misconceptions about TB were widespread compared to the younger generations who most probably have experienced more information, education communication (IEC) messages on TB.

The findings presented here point to significant differences in myths and misconceptions by ethnic background. While it is difficult to tease out specific triggers of these differences, cultural epidemiologists [29,30] seem to agree that the interpretations and conceptions of some diseases can be shaped by the views of the dominant group. For instance, among Akans in Ghana, there is a belief that TB could arise from ancestral punishment due to lack of care provided to family members who have died from TB, hence the name *Nsaman wa* (ghost cough). In the Volta Region of Ghana too, TB is known as *yomokpe* (grave yard), suggesting that once infected, death was unavoidable. These myths and misconceptions might have occasioned the ethnic differences.

Our results reported here have implications for national as well as sub-national health education policy design and implementation. Although formal education and media channels (print, radio and television) remain important channels for communicating health messages, we did not find evidence of their impact on myths and misconceptions about TB transmission in the present study. It is possible that IEC issues on the routes of TB transmission could have been taken for granted. The fact that respondents with higher formal education were more likely to accept misconceptions than those with low formal education points to the need to integrate TB issues into curriculum of formal education subjects such Social Studies. Already, HIV/AIDS, a disease that shares close synergies with TB forms an integral component of Social Studies course content at the basic and secondary levels, and runs as a full model in some universities, colleges and polytechnics. This presents opportunities for fusing TB and HIV/AIDS modules, which is likely to reduce myths and misconceptions about the later. Current content of IEC messages on TB in the country disproportionately focus on how to prevent infection through cough etiquettes to the neglect of myths and misconceptions.

The fact that high exposure to all the media included in this analysis did not correspond to low misconceptions about TB transmission could be an indication of their ineffectiveness in reaching out to the majority of the population that requires such information. It therefore portends that other channels such as using religious gatherings and community durbars could be other alternatives that can be exploited for TB-related IEC.

Some limitations of the study should be taken into account. First, the questions used to measure myths and misconceptions as captured in the 2008 GDHS were adopted from questions that have been extensively used to measure myths and misconceptions about HIV and their validity are not in doubt [31]. However, it must be borne in mind that the “truthfulness” of responses provided by respondents in a survey like the DHS is difficult to ascertain. Several factors such as respondents not paying significant attention to the questions, or on a particular aspect of the questionnaire, could compromise the ‘quality’ of responses that were provided [32-34]. Nonetheless, ICF Macro International in collaboration with local data collection agencies has taken measures to improve the reliability and validity of findings obtained from the DHS. Some of these measures include the translation of questionnaires into local languages coupled with the use of field assistants who are competently fluent in the local languages of localities that were sampled into the survey. Although not entirely related, a multi-country study that examined sexual behaviour using DHS data found high levels of validity of the measures of sexual behaviour. These observations point to minimal problems associated with the validity of responses generated from the DHS [35]. A recent analysis of some popular questions that are used to measure misconceptions about HIV, which have been adopted for TB, also found positive indications about validity [36]. Second, our use of a cross sectional data limits us to make any causal claims, thus, does not allow deeper understanding into the issues we have analysed in this paper. What we have presented in this article only points to associations. The nationally representative nature of the survey also provides indications about populations who require attention in IEC meant to reduce myths and misconceptions relative to TB transmission. Characteristic of knowledge, attitudes and practices (KAP) survey data, the study affords us insights into identifying some spatial, socioeconomic and demographic features of populations who can be targeted with educational messages in respect of myths and misconceptions about TB transmission.

## Conclusion

This study examined some key drivers of myths and misconceptions about TB transmission using a representative sample of Ghanaians between 15 and 59 years. Myths and misconceptions persist in the general population but this varies by spatial, socioeconomic, and demographic characteristics. Particularly, wide significant spatial differences in myths and misconceptions are observed. It is therefore recommended that behavioural change communication messages on TB should be segmented based on individual (for instance, education, sex) and communal (e.g. region of residence, urban–rural) characteristics so as to make the greatest impacts.

## Competing interests

The authors declare that they have no competing interests.

## Authors’ contributions

Concept and data analysis – JAA: First draft – JAA: AKK reviewed the paper for critical intellectual contribution. All authors approved the manuscript for submission.

## Pre-publication history

The pre-publication history for this paper can be accessed here:

http://www.biomedcentral.com/1472-698X/13/38/prepub
